# Magdeburger Arbeitsmedizin feiert 50. Geburtstag

**DOI:** 10.1007/s40664-022-00478-6

**Published:** 2022-08-17

**Authors:** Beatrice Thielmann

**Affiliations:** Bereich Arbeitsmedizin, Medizinische Fakultät, Otto-von-Guericke-Universität Magdeburg, Leipziger Str. 44, 39120 Magdeburg, Deutschland

**Keywords:** Historie, Forschung, Sachsen-Anhalt, Universitätsmedizin, Betriebsmedizin, History, Research, Saxony-Anhalt, University Medicine, Occupational Medicine

## Abstract

Die universitäre Arbeitsmedizin in Magdeburg feiert 50. Jubiläum. Dieser Artikel befasst sich mit den Forschungsaktivitäten des Instituts seit der Übernahme der Leitung durch Prof. Böckelmann und stellt die Tätigkeiten der Mitarbeiter und der Gastwissenschaftler dar.

## Abstract (en)

The academic Occupational Medicine in Magdeburg celebrates its 50th anniversary. This article deals with the research activities of the institute since Prof. Böckelmann became director. The paper presents the activities of the institute’s staff and guest researchers since 2008.

Das arbeitsmedizinische Institut (IAM, Abb. 1) der Otto-von-Guericke-Universität (OVGU) feiert nachträglich am 2. September 2022 sein Jubiläum anlässlich des 50. Geburtstags am 1. Juni 2022.

**Figure 1:**
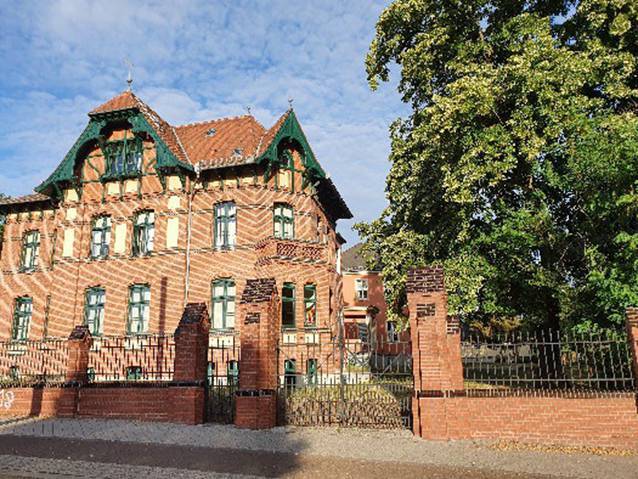

Obwohl die Geschichte der Arbeitsmedizin in der Landeshauptstadt Magdeburg bereits 1927 in Form einer Beratungsstelle für Berufskrankheiten begann, erfolgte die Gründung des arbeitsmedizinischen Instituts, damals als „Abteilung Arbeitshygiene“ an der damaligen Medizinischen Akademie Magdeburg, erst am 1. Juni 1972. Die ehemaligen Institutsleiter und deren Forschungsschwerpunkte sind in Tab. 1 aufgeführt. Die studentische Lehre und der Aufbau der Institutsambulanz werden hierbei nicht weiter betrachtet. Beides erfolgte sukzessive unter Mitwirkung aller Institutsleiter und der -leiterin.

**Table 1:** 

Zeitraum	Leiter	Forschungsschwerpunkte
06/1972–1985	Prof Dr. P.-J. Reum	Arbeitsmedizinische, arbeitsphysiologische und arbeitshygienische Untersuchungen auf den Binnenschiffen und bei den Beschäftigten der Binnenreederei der DDR
1985–1992	Prof. B. Hartmann	Arbeitsmedizinische Jugendforschung (Fortführung Prof. Pfister), Belastung und Beanspruchung des Stütz- und Bewegungssystems bei verschiedenen Arbeitsformen, Implementierung psychophysiologischer Methoden
1993–2008	Prof. E. Pfister	Kooperation im Rahmen des BMBF-geförderten Magdeburger Neuroverbundes bei vorrangiger Klärung der Problemstellung fraglicher neurotoxischer Spätschäden bei Beschäftigten der Chemischen Industrie, der Buntmetallurgie, von Autolackierereien und Druckereien (Fortführung Prof. Böckelmann)

Seit dem 01.10.2008 übernahm die Leitung PD Dr. med. habil. Irina Böckelmann. Hochschulpolitisch motiviert, erfolgte zeitgleich die Umbenennung des Instituts in den „Bereich Arbeitsmedizin“. Dr. Böckelmann habilitierte bereits 2006 mit dem Thema „Arbeitsmedizinische Fragen zur Neurotoxizität beruflicher Blei- und Lösemittelexposition“, wurde zur Privatdozentin ernannt und erhielt die Venia legendi für das Fach „Arbeitsphysiologie“. Im Jahr 2012 wurde ihr die außerplanmäßige Professur verliehen.

Im Andenken an Professor Dr. med. Dr. phil. Joseph Rutenfranz (1928–1989) verlieh die Deutsche Gesellschaft für Arbeitsmedizin und Umweltmedizin e. V. (DGAUM) die Joseph-Rutenfranz-Medaille. Am 07. März 2018 wurde diese besondere Auszeichnung im Rahmen der Jahrestagung der DGAUM an Professorin Böckelmann in München vergeben. Dafür ausschlaggebend waren „ihre besonderen wissenschaftlichen Leistungen und die fachpolitischen Verdienste in der Arbeitsphysiologie“, was zahlreiche drittmittelfinanzierte Forschungsprojekte, rege Publikationstätigkeit und ihr Engagement im Forum Arbeitsphysiologie in der Vergangenheit bis heute bestätigen. Auswahlweise zu nennen sind die drei BMBF-geförderten Projekte: AVILUS-Projekt – Nutzerbezogene Entwicklung und Untersuchung AR-basierter Werkerassistenzsysteme, 3D-basierte Assistenztechnologien für variantenreiche Montageprozesse – Menschzentrierter Arbeitsplatz der Zukunft („3D-Montageassistent“) als Teilprojekt im BMBF-Verbund, Gesundes mobiles Arbeiten mit digitalen Assistenzsystemen im technischen Service (ArdiAS) im BMBF-Forschungsschwerpunkt „Arbeit in der digitalisierten Welt“ im Rahmen des FuE-Programms „Zukunft der Arbeit“ als Teil des Dachprogramms „Innovationen für die Produktion, Dienstleistung und Arbeit von morgen“.

Aktuelles Forschungsprojekt ist eine von der BG für Gesundheitsdienst und Wohlfahrtspflege (BGW) geförderte Studie: „Ursachen und Folgen psychischer Belastung im Arbeitsalltag und im Notdienst der Tierärzteschaft in der Bundesrepublik Deutschland“ als Förderung (Nr. 1544).

Daneben werden in Magdeburg noch zahlreiche hausmittelfinanzierte Forschungsprojekte durchgeführt, wie beispielsweise:

- Belastungssituationen und deren Beanspruchungsfolgen im Arbeitsalltag von Beschäftigten in der Krankenpflege der ZNA der Universitätsklinik Magdeburg,
- Studie zu Belastungen und Beanspruchung von Einsatzkräften des Rettungsdienstes (inkl. Notärzte) und der Leitstellen während der Pandemie (aktuell 4 Querschnitte),
- Einfluss der Bildschirmzeit im Semesterverlauf auf die Schlafqualität Studierender der Hochschule Magdeburg-Stendal,
- Herzratenvariabilität unter besonderer Berücksichtigung der objektiven Stimmfunktion,
- Auswirkungen besonderer Arbeitsformen auf die Gesundheit der Arbeitnehmer – ein Vergleich von Akkord- und Fließarbeit.

Erwähnenswert ist auch die Erarbeitung und die erneute Aktualisierung der AWMF S2k-Leitlinie „Nutzung der Herzschlagfrequenz und der Herzfrequenzvariabilität in der Arbeitsmedizin und der Arbeitswissenschaft“ unter Magdeburger Federführung von Prof. Dr. Böckelmann und PD Dr. Sammito und unter Mitwirkung von Dr. B. Thielmann.

Der Magdeburger Bereich Arbeitsmedizin hat in der 25-jährigen Geschichte des Forums Arbeitsphysiologie vier Symposien Arbeitsmedizin und Arbeitswissenschaft für Nachwuchswissenschaftler:innen aus Deutschland, Österreich und der Schweiz veranstaltet. Diese Symposien in den Jahren 1999, 2007, 2014 und 2019 waren stets sehr gut besucht, und die Magdeburger Nachwuchswissenschaftlergruppe war zahlreich vertreten.

Prof. Böckelmann gehört zu den Mitorganisator:innen des 7. Internationalen Symposiums zur Herzfrequenzvariabilität (HRV) „Methoden und Anwendungen in Sportwissenschaft, Arbeits- und Intensivmedizin sowie Kardiologie“ (2017) sowie des 8. Internationalen HRV-Symposiums „Herzfrequenzvariabilität: Anwendungen in Forschung und Praxis“ (2020) und zu den Herausgeber:innen der beiden Buchbände zu diesen HRV-Symposien.

Zudem richtete das IAM den diesjährigen 68. Frühjahrskongress der Gesellschaft für Arbeitswissenschaft (GfA) als dreitägige Online-Veranstaltung vom 2. bis 4. März 2022 neben den Professuren für Arbeitswissenschaft und für Betriebspädagogik der OVGU und dem Fraunhofer Institut für Fabrikbetrieb und -automatisierung Magdeburg aus.

Gemäß dem Motto „Lieber mittendrin als nur dabei“ wirkt Prof. Böckelmann gern auch als „Pilotprobandin“, um die Windkraftanlage auf 104 m Höhe von innen zu erkunden (Abb. 2) oder stellt ihre Forschungsthemen den Kindern im Rahmen des Programms „Ferienfreizeit“ des Familienbüros der OVGU kindgerecht vor (Bildercollage Abb. 3), sodass Kinder im Alter zwischen dem 8. und 12. Lebensjahr die Farbwahrnehmung entdecken und selbst erforschen konnten.

**Figure 2:**
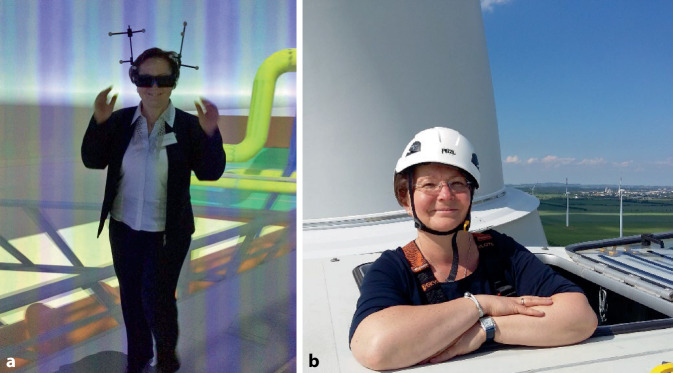

**Figure 3:**
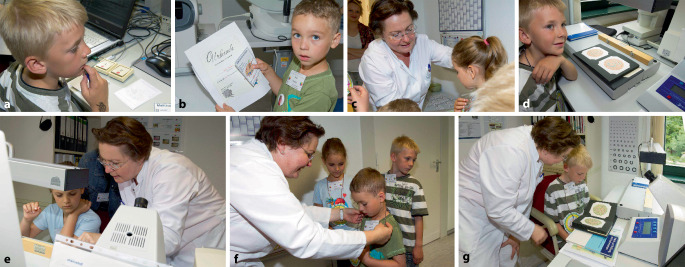

Neben der Forschung und Lehre gehören die Klinische Arbeitsmedizin und Dienstleistung im Zusammenhang mit den regelmäßigen hygienischen Überprüfungen sämtlicher OP-Bereiche innerhalb der Universitätsmedizin sowie die Untersuchung der Luftreinheitsbedingungen (Reinheits-Klassen A, B und C) im Bereich der Blutverarbeitung und in der Apotheke zu weiteren Kernaufgaben des IAM. Drei haushaltsfinanzierte ärztliche und wissenschaftliche Mitarbeiterinnen (Prof. Irina Böckelmann, Dr. Sabine Darius und OÄ Dr. Franziska Heinemann) und ein drittmittelfinanzierter wissenschaftlicher Mitarbeiter (Dr. cand. R. Pohl) werden von drei Assistentinnen und einer Sekretärin unterstützt.

Bis dato zeichnet sich das IAM durch eine präventiv und interdisziplinär vernetzte Expertise aus, welche insbesondere durch die Gastwissenschaftler:innen geprägt und gestützt wird.

Als Gastwissenschaftler und in der Lehre unterstützend tätig ist PD Dr. med. Stefan Sammito, der 2017 ebenfalls an der Medizinischen Fakultät der OVGU habilitierte mit dem Thema „Alters- und geschlechtsabhängige Referenzwerte für die Herzfrequenzvariabilität“. Für seine besonderen Verdienste auf dem Gebiet der Arbeitsphysiologie („Experimentelle flugmedizinische Forschung“ am Zentrum für Luft- und Raumfahrtmedizin der Luftwaffe) wurde er 2021 mit der oben erwähnten Joseph-Rutenfranz-Medaille ausgezeichnet. Ebenfalls langjährig als Gastwissenschaftler:innen am IAM wirkend sind Dr. med. B. Thielmann und Dr. rer. medic. H. Schumann, die Anfang 2022 die AG „Rettungsdienstforschung“ gründeten, welche sich aktuell mit den Belastungen und der Beanspruchung von Einsatzkräften des Rettungsdienstes und der Leitstellen während der Pandemie beschäftigen. Diese Doppelführung wird als Vorteil angesehen, um die Rettungsdienstforschung langjährig zu etablieren. Daneben ist Dr. B. Thielmann bei weiteren Forschungsprojekten involviert. Dr. cand. R. Bölsch-Peterka erreichte mit ihrer Publikation „Durchführung von digitalen Arbeitssituationsanalysen für die mobil-flexible Arbeit zur Erhebung von psychischen Belastungsfolgen“ im *Zentralblatt für Arbeitsmedizin, Arbeitsschutz und Ergonomie* jüngst regional mediales Interesse und führte sogar ein Interview mit dem MDR. Die Gastwissenschaftler Dres. rer. medic. D. Kirchhoff und F. Kirsch sowie Dr. cand. M. Krowicki unterstützen das Team mit ihrer Expertise aus dem Gesundheitswesen. Weiterhin anzumerken sind die forschungsbasierten Kooperationen mit der Hochschule Magdeburg-Stendal und dem Fraunhofer-Institut für Fabrikbetrieb und -automatisierung Magdeburg. Zudem bestehen nationale auch internationale Kooperationen wie bspw. mit der Charkower Nationalen Medizinischen Universität (Prof. I. Zavgorodnij). Neben Prof. Zavgorodnii hospitierte Dr. cand. Håvard R. Karlsen vom Department of Psychology der Norwegian University of Science and Technology zu Beginn der SARS-CoV-2-Pandemie am IAM. Hieraus ergaben sich jeweils mehrere internationale Publikationen.

Im Bereich Arbeitsmedizin wurden in den letzten 10 Jahren eine Habilitation (Dr. Stefan Sammito) und 38 Promotionen erfolgreich verteidigt. Durch die enge Kooperation mit der Hochschule Magdeburg-Stendal entstanden weitere 18 Studien im Rahmen der Bachelor- und Masterarbeiten. Beachtenswert ist auch die Veröffentlichung von 124 Originalarbeiten, 98 Übersichtsarbeiten, 169 Buch‑/Kongressbeiträgen und die Durchführung von 237 Vorträgen und Postern im letzten Jahrzehnt.

Das IAM lässt sich zusammenfassend beschreiben als jung, dynamisch, flexibel, interdisziplinär vernetzt und erfolgreich. Nicht unverdient feiert das IAM nunmehr den 50. Geburtstag, welcher als hybride Veranstaltung am 02.09.2022 geplant ist. Der wissenschaftliche Teil (Infobox) veranschaulicht wunderbar die Interdisziplinarität des Teams, welches durch einen Gastvortrag von Prof. B. Hartmann abgerundet wird.

HAPPY BIRHTDAY!

## Infobox Wissenschaftliches Programm der Jubiläumsfeier

- Prof. I. Böckelmann „50 Jahre Arbeitsmedizin in Magdeburg“
- PD Dr. S. Sammito „Flight Safety – der Beitrag der flugphysiologischen Forschung zur Sicherheit in der Flugmedizin“
- Prof. Hartmann „Arbeitsphysiologie: Mit dem Wandel der Arbeitsfeld Schritt halten“
- Dr. B. Thielmann „Psychokardiologie: Ihre Stellung in der Arbeitsmedizin“
- Dr. cand. R. Bölsch-Peterka „Diversität in der Arbeitsmedizin – Impulse, die uns zukünftig begleiten könnten“
- Dr. Schumann „Arbeitsmedizin, Rettungsdienst & Forschung? From Silence to Voice!“
- Dr. F. Heinemann „Der Betriebsarzt in Zeiten der Pandemie“
- Dr. cand. R. Pohl „Psychische Folgen der Corona-Pandemie im Arbeitsleben“
- Dr. S. Darius „Psychische Belastungen in der heutigen Arbeitswelt – wie gehen wir damit um“

